# Mania as an unique life event

**DOI:** 10.4103/0019-5545.37672

**Published:** 2007

**Authors:** Manu Arora, Piyali Mukherjee, Samir Kumar Praharaj, Vinod Kumar Sinha

**Affiliations:** Central Institute of Psychiatry, Kanke, Ranchi, Jharkhand - 834 006, India; * Ranchi Institute of Neuropsychiatry and Allied Sciences, Kanke, Ranchi, Jharkhand - 834 006, India

**Keywords:** Mania, life event, genetic anticipation

## Abstract

Despite substantial evidence of relationship between life events and psychiatric illness in general and mood disorder in specific, none of the research has documented ‘mania’ itself as a life event in the onset of mania in a family member who is genetically vulnerable to the disorder. We describe three such cases that had strong temporal association between manic episode in the family member and subsequent manic episodes among the first-degree relatives. Mania as a life event might play a significant role as a precipitator towards the onset of mood disorder with a special impact on those who have vulnerability towards the disorder; the cases are discussed from both psychological and biological perspective.

Life event is a relatively abrupt change in the external environment, involving mostly the social element.[1] Unlike the chronic stresses that are concerned with ongoing major role-related issues or minor irritations of daily hassles, life events are discrete, observable stressors.[2] Moreover, it has generalized effects and is expressed in varying symptomatology depending upon the individual's characteristics.[3] It has been suggested through research that the first episode of mania is more likely to be triggered by life event unlike in the later episodes.[4] In the following case series, the importance of psychiatric illness (mania) precipitating an episode of mania in genetically predisposed individuals is highlighted.

## CASE REPORTS

### Case 1

A 21-year-old unmarried male, without any past or family history of psychiatric disorder, was diagnosed as having moderate depression and was prescribed escitalopram 20 mg/day. After 1.5 months of treatment, he switched into mania, characterized by increased psychomotor activity, euphoric mood and grandiosity along with demanding behaviour. His diagnosis was changed to bipolar affective disorder, current episode mania without psychotic symptoms. Escitalopram was discontinued and he was put on olanzapine 10 mg and oxcarbazepine 600 mg, which was increased to 1200 mg per day; subsequently his manic symptoms improved. However, during the course of illness, the extremes of behavior disrupted his lifestyle; he became negligent towards his domestic and social responsibilities. Being a student of graduation, his education suffered a lot inspite of being hard-working prior to illness; soon he lost contact with his friends. His parents became very worried and concerned for their only child. They were scared as he would be very abusive at any slightest provocation. They would feel embarrassed to talk about their son to anyone who would enquire about him. Three months later, his mother, who was 43 years old, without any previous history of psychiatric disorder, had an acute onset of illness comprising of increased talk, elated mood, excessive religiosity and inclination towards good dresses and food. She was diagnosed as mania without psychotic symptoms, and showed good response to lithium 1050 mg/day.

### Case 2

A 24-year-old married male, presented with complaints of talkativeness, making grandiose claims, abusive and assaultive, increased spending of money and decreased need for sleep; he was diagnosed as mania with psychotic symptoms. He had no past history of psychiatric illness but had a family history of bipolar affective disorder in his paternal uncle. Prior to his illness, he had a happy marital life. At the onset of illness, he neglected self-care as well as his wife and child. His occupational life also suffered as he discontinued work, which strained the economic condition of the family. The stressful home environment affected everyone in the family including his parents. They remained anxious, sad and spent sleepless nights in taking care of their son and blamed themselves for his illness. Fifteen days later, his father, who was 42 years old, had an acute onset of similar symptoms and was diagnosed as mania with psychotic symptoms. Both of them required hospitalization and improved simultaneously on lithium 900 mg and olanzapine 15 mg per day.

### Case 3

A 46-year-old married male, developed manic episode over 2 weeks with irritability, decreased need for sleep, aggressive behaviour, hallucinatory behavior and delusions of grandiosity, claiming to be ‘Lord Shiva’ with special power to save the mankind. He had a history of depression 25 years back. His son and wife were extremely worried, because his grandiose claim of being God caught the attention of people in their neighbourhood. With such a commotion prevailing in their house, 7 days later, his son, who was 22 years old, developed irritability, decreased need for sleep, abusive behaviour and similar grandiose claims. Both father and son had to be admitted to the hospital; they improved on lithium 900 mg/day and aripiprazole 15 mg/day. After discharge from hospital, they were maintaining well at follow up after 3 months.

## DISCUSSION

In all the three cases, manic episodes occurred in the first-degree relatives shortly after the onset of mania in the first member. There was a temporal association between the stressor (i.e. manic episode in the family member) and subsequent reaction (i.e. manic episode in the first-degree relative); the duration varied between 1 and 6 weeks. The reason to perceive illness as a stressor could be that it was their first experience of mental illness in the family.

A manic episode has not been previously reported as a life event leading to precipitate an episode of mood disorder. Johnson[5] grouped life events in bipolar disorder under negative life events, schedule-disrupting events and goal-attainment events. In our series, schedule disruption in family members could be assumed to have led to the onset of manic episodes. According to this model, either sleep disruption[6] or more generally schedule disruption[7] might be the mechanism through which life stressors lead to the onset of manic episodes. Alternatively, the episode of mania in a family member might have been perceived as a ‘loss’ to the first-degree relatives who are emotionally bonded to the patient. Drawing a parallel with the psychodynamic model focussing on ‘manic defenses’ in which mania is perceived as a flight from painful feelings that occur after negative events,[8] the manic episode that occurred in the first-degree relatives can be seen as a symbol to ‘denial of loss’. These assumptions are, however, supported by the reports of ‘funeral mania’, where manic symptoms have been demonstrated in people at funeral or death of close relatives.[9,10] Moreover, cognitive behavioural formulations view people with bipolar disorder who might avoid focussing on threatening information.[11] In addition, psychological defense mechanism of ‘identification with the deceased’ was reflected in one of our cases, which showed sharing of similar grandiose delusion of being God in both father and son. Another observation was that the onset of manic episode was at a younger age in the offspring as compared to that of the parents. This may be attributed to genetic anticipation, i.e. a decreasing age at onset and/or increasing disease severity in successive generations due to the increase in number of trinucleotide repeats.[12]

To conclude, mania as a life event might play a significant role as a precipitator towards the onset of mood disorder with a special impact on those who have genetic vulnerability. Further research is required in this area to highlight the importance of specific life events determining the onset and course of illness and its influence on specific symptoms of the disorder. In addition, understanding the nature and importance of life events in bipolar disorder could help in the prevention of further episodes.
